# Management of the poultry red mite *Dermanyssus gallinae* with physical control methods by inorganic material and future perspectives

**DOI:** 10.1016/j.psj.2023.102772

**Published:** 2023-05-05

**Authors:** Ee Taek Hwang

**Affiliations:** Department of Food Biotechnology, Dong-A University, Busan 49315, Republic of Korea

**Keywords:** *Dermanyssus gallinae*, physical control of poultry red mite, inorganic materials, mite infestations

## Abstract

Poultry red mite (**PRM**), the ectoparasitic mite *Dermanyssus gallinae* found in laying hen farms, is a significant threat to poultry production and human health worldwide. It is a suspected disease vector and attacks hosts’ other than chickens, including humans, and its economic importance has increased greatly. Different strategies to control PRM have been widely tested and investigated. In principle, several synthetic pesticides have been applied to control PRM. However, recent alternative control methods to avoid the side effects of pesticides have been introduced, although many remain in the early stage of commercialization. In particular, advances in material science have made various materials more affordable as alternatives for controlling PRM through physical interactions between PRM. This review provides a summary of PRM infestation, and then includes a discussion and comparison of different conventional approaches: 1) organic substances, 2) biological approaches, and 3) physical inorganic material treatment. The advantages of inorganic materials are discussed in detail, including the classification of materials, as well as the physical mechanism-induced effect on PRM. In this review, we also consider the perspective of using several synthetic inorganic materials to suggest novel strategies for improved monitoring and better information regarding treatment interventions.

## INTRODUCTION

**Figure 1:**
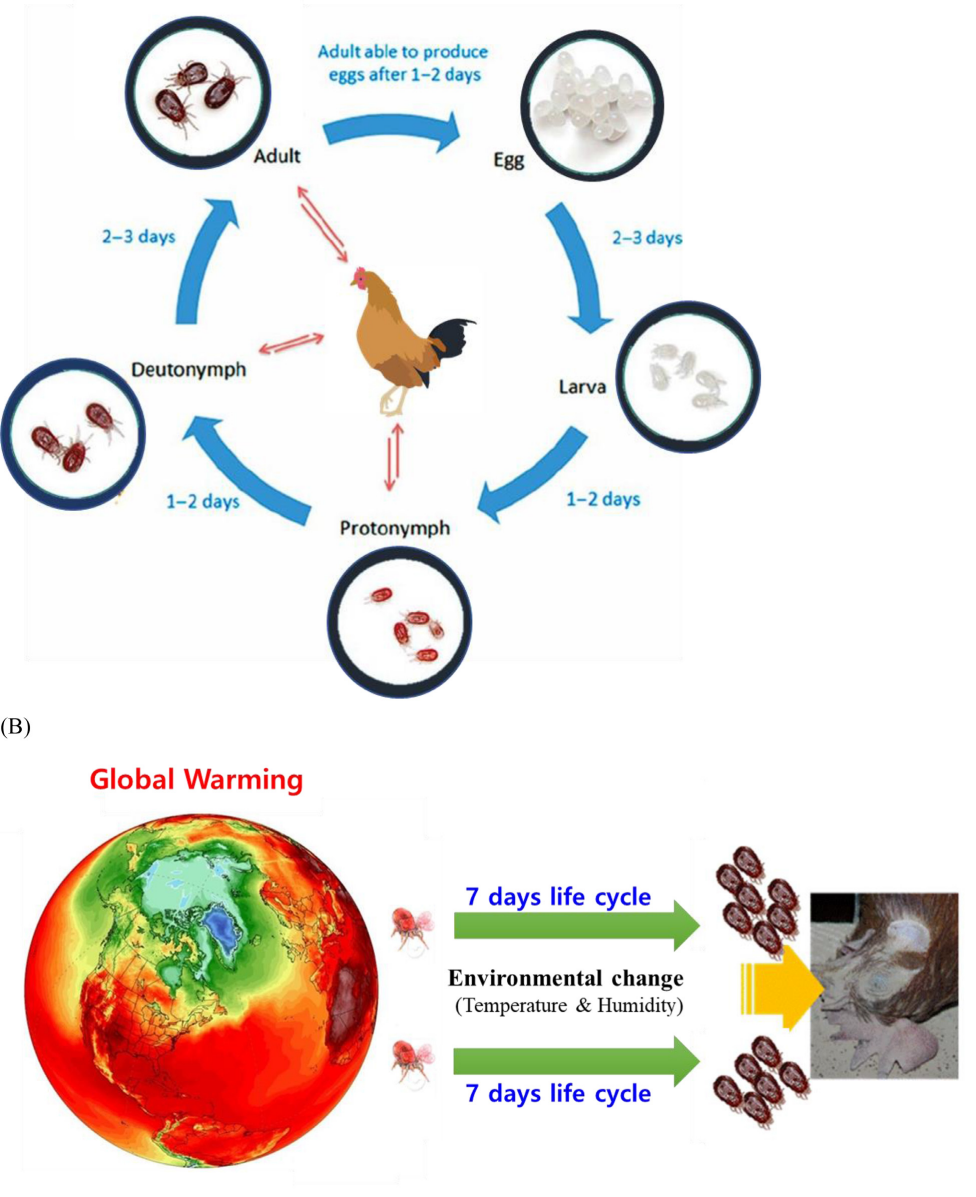
(A) Schematic illustration of the life cycle of the poultry red mite (PRM), *Dermanyssus gallinae*, under favorable conditions and (B) recent worsening chicken mite problems due to climate change (abnormal high temperatures and humidity), which has prolonged the PRM breeding season.

*Dermanyssus gallinae*, also known as the poultry red mite (**PRM**), is an ectoparasitic mite found in laying hen farms worldwide and is a major pest in the poultry industry (Chauve, 1998). During the daytime, PRM normally hide in cracks and crevices in the poultry house and then come out to feed on poultry at night. The mite feeds on the blood of hens in only 30 to 60 min (Koenraadt and Dicke, 2010b). The mite induces severe stress on hen health and welfare by causing anemia. An adult female PRM is approximately 1 mm in length and 0.4 mm in width, and adult colors range from gray to red depending on engorgement. The mite has 5 life stages: egg, larva, protonymph, deutonymph, and adult (Figure 1) (George et al., 2014). Its life cycle can be completed within 7 d under favorable conditions, with rapid population growth. A blood meal is required for molting from the protonymph to the deutonymph to the adult (Kilpinen, 2001).

**Figure 2:**
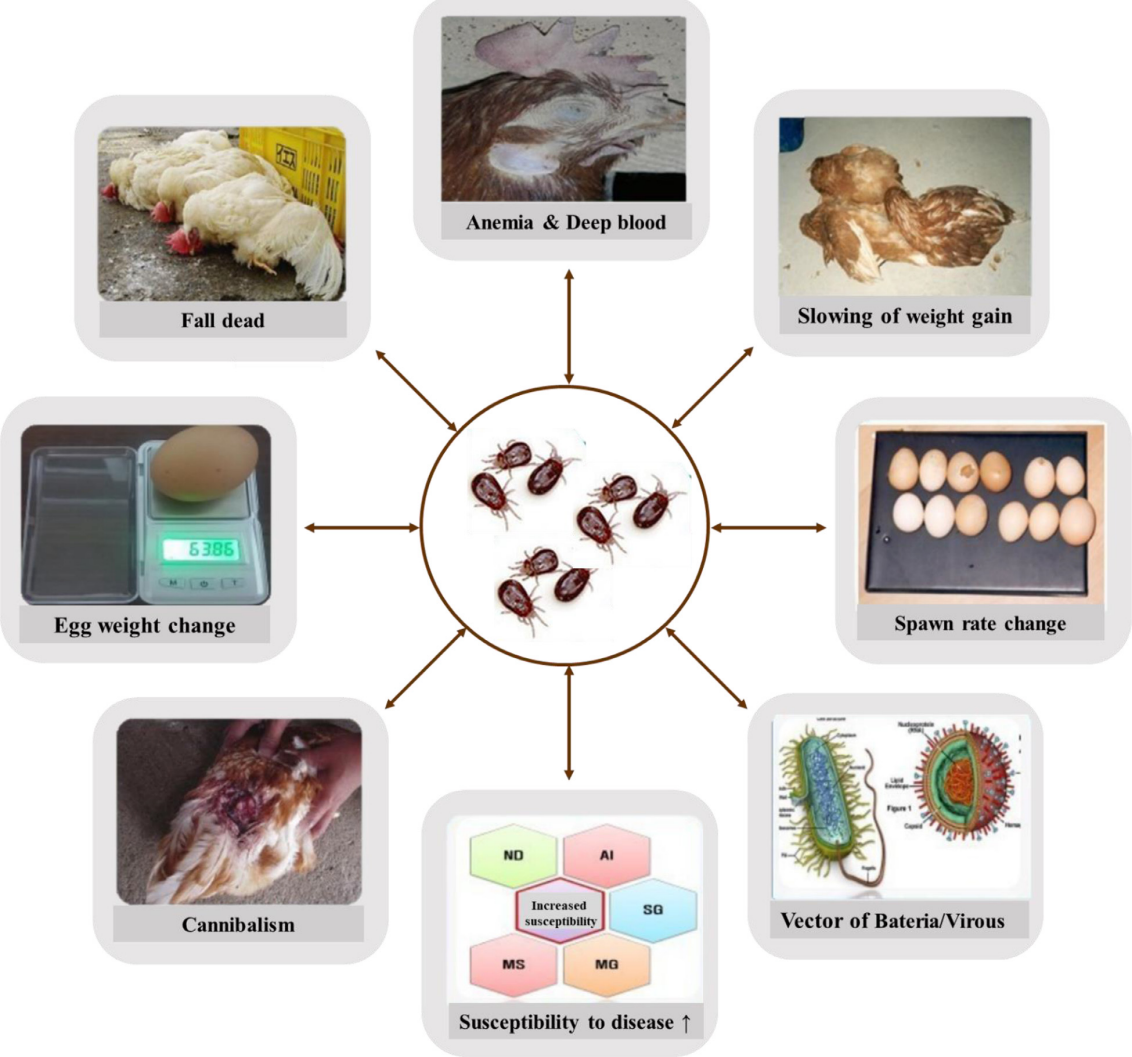
Schematic illustration of the economic, environmental, and health issues induced by mite infestations.

Mite infestations are a problem for humans and hens, inducing reductions in egg production, feed conversion efficiency, body weight gains, and egg size, and also causing significant animal health problems such as increased mortality, stress, weight loss, anemia, and compromised immunity (Figure 2) (Beugnet et al., 1997). The mite may act as a vector for a number of pathogenic poultry infections that spread diseases caused by bacteria and viruses (Chirico et al., 2003). Several incidents of PRM parasitizing humans have been recorded when humans are in contact with the mite while working. When bitten, mild irritation, skin lesions, and dermatitis have occurred as minor conditions. However, as the primary characteristic of PRM is that it is an obligatory blood-sucking parasite (Nordenfors et al., 1999; Bruneau et al., 2003), depending on the level of infestation, it can cause increased mortality and behavioral disorders owing to sleep deprivation (Kilpinen et al., 2005; Sigognault Flochlay et al., 2017). Economic losses from poultry mite infestations severely affect the productivity in the egg-laying industry. The mite has a worldwide distribution and causes serious problems in European farms. Egg production is affected by reduced growth rate, egg production, and egg quality (Sigognault Flochlay et al., 2017). For example, a caged housing system study showed that mortality ranged from 1 to 4% in units owing to parasitism by PRM, and egg production was reduced by 10% (Wójcik et al., 2000). The estimated cost of PRM infestation as a result of mite control and production losses is €130 million annually (Sparagano et al., 2009). Recently, de Jong and van Emous (2017) estimated that the current total cost of red mite infestation is €0.60 per hen per yr in the Netherlands, which represents an increase of approximately 40% compared with 2005 for the total cost of mite control.

In addition to economic losses, mite infestations cause major physiological damage in animals. According to previous studies, productivity losses can reach from €0.57 to €2.50 per hen per yr (Kilpinen, 2005). In Europe alone, the approximate total annual cost is €360 million, with more than 300 million hens suffering from infestations, resulting in severe economic losses in the egg production industry, including health and welfare issues in the hens (Axtell, 1999; Aulakh et al., 2006; Arkle et al., 2008).

The scientific control of PRM has been described in the literature for over 20 yr (Chauve, 1998; Sparagano et al., 2014; George et al., 2015). Currently, the main treatments available against PRM are synthetic neurotoxic acaricides such as organophosphates and pyrethroids (Abbas et al., 2014). The oral administration of systemic ectoparasiticides, such as fluralaner, was recently approved in hens (Huyghe et al., 2017). However, significant restrictions for synthetic acaricide use have been imposed because of their negative impact on both human health and the environment, and some neurotoxic substances may be carcinogenic or impair mental health (Ansari et al., 2014). In addition, veterinary pesticides may be transferred to field crops through organic manure in the form of poultry litter (Kaczala and Blum, 2016). To date, chemical treatments have traditionally been used to control PRM; however, there are a limited number of acaricides authorized to treat mite infestations (Yuan et al., 2016). In addition, resistance to commonly used acaricides reduces their efficiency (Marangi et al., 2009). The classical use of chemical pesticides for mite treatment in veterinary medicine presents a multitude of problematic challenges, including the ineffectiveness of active ingredients, mite resistance, undesirable residues in the environment, and unacceptable risks to nontarget organisms (Coles and Dryden, 2014). To resolve these issues, alternative strategies are required to overcome the disadvantages of existing chemical-based treatments.

Among the alternative treatments available, botanicals (also known as natural pesticides or phytochemicals) represent an alternative control strategy that is less harmful to the environment and human health (Isman, 2006). The substances derived from these natural products can be grouped into 2 categories based on their uses: acaricides, which are toxic to the pest, and repellents, which deter the pest (Veeresham, 2012; Soulié et al., 2021). Previous studies have evaluated the potential of plant-based products as acaricides and repellents of PRM (George et al., 2009b). These can interfere in the chemical interactions between a pest and its host by 1) forming an odor barrier to prevent the pest from entering a space occupied by the host, or 2) preventing pests from developing under favorable conditions on the target host (Ben-Issa et al., 2017a, b). The use of plant-derived secondary metabolites as repellents is an interesting alternative to synthetic acaricides for PRM. They have low environmental persistence and natural degradation pathways, and even mitigate the impact of pest management on biodiversity in agriculture (Lushchak et al., 2018). However, the effective application of these for pest control requires an improved understanding of their mechanisms and detailed toxicity studies owing to their highly toxic properties, even at low concentrations, which depends on the plant (Deletre et al., 2016). Vaccination is also a novel, environmentally friendly, and promising alternative for PRM control with many advantages, such as reduced pesticide use and resistance unlikely, and no contamination of the environment and animal products (Lushchak et al., 2018; Lima-Barbero et al., 2019). Recombinant proteins have been used to immunize host animals against parasitic species. For example, the Bm86 recombinant protein is a commercial vaccine that can protect cattle from *Boophilus microplus* tick infestations (Fuente et al., 2007). However, vaccine development for PRM is challenging owing to limited information about its constituent proteins (Bartley et al., 2012; Makert et al., 2016). Therefore, recent research has focused on exploring alternative biological controls of PRM, including natural enemies, entomopathogenic fungi, nematodes, bacterial endosymbionts, semiochemicals, and growth regulators (Sparagano et al., 2014). Accordingly, a range of PRM control methods have been developed, including organic substances such as synthetic pesticides, pheromones, essential oils, plant-derived compounds, vaccines, and biological approaches. Additionally, alternative methods, such as inert dust or minerals and disinfectants, have been used inside poultry facilities without prolonged residual toxicities (Soulié et al., 2021).

In this review, we provide an overview of the available knowledge on different types of PRM control. First, we introduce PRM infestation, with descriptions of the clinical effects of mite infestation, the role of PRM as a disease vector in public health and zoonotic diseases, and human medicine. Second, we summarize the conventional control of PRM using organic and inorganic methods. To focus on the scope of the review, we discuss in detail the physical control of PRM in terms of experiments done to progress the implementation of inorganic materials in PRM management. Finally, future perspectives for inorganic material selection exerted by mechanism-based behavior of PRM are given, which can be applied as potential sustainable treatment alternatives without any toxicity.

## POULTRY RED MITE INFESTATION

### Clinical Effects and Role as a Disease Vector

Of clinical concern is the severity of the effects induced by PRM parasitism on bird health and welfare. The first clinical sign in infested animals is subacute anemia from repeated mite bites (Sigognault Flochlay et al., 2017). A laying hen can lose more than 3% of its blood volume a night. In extreme cases, PRM infestation can cause hens to die from severe anemia (Sigognault Flochlay et al., 2017). Besides this direct effect of hematophagous parasitism, PRM is also a vector of several bacterial and viral pathogens in mammals (including humans) and birds. These include the paramyxovirus that causes Newcastle disease, the Eastern, Western, and Venezuelan equine encephalomyelitis viruses, avian influenza A virus, and bacteria such as *Escherichia coli, Erysipelothrix rhusiopathiae, Pasteurella multocida, Salmonella gallinarum*, and *S. enteritidis* (De Luna et al., 2008; Valiente Moro et al., 2009; Moro et al., 2010; Sommer et al., 2016; Sigognault Flochlay et al., 2017). Poultry mites often serve as long-term hosts for viral and bacterial pathogens, thus becoming reservoirs of these pathogens (George et al., 2015). For example, the eastern equine encephalomyelitis virus and *P. multocida* were isolated from mites 30 d and 2 mo after the ingestion of blood meals from infected chickens, respectively (De Luna et al., 2008).

### Role of PRM in the Transmission of Zoonotic Diseases

In addition to affecting chicken health, PRM infestations also pose public health concerns due to the role of the mite as a zoonotic disease vector, with medical impacts on humans (Sigognault Flochlay et al., 2017). As described above, PRM is involved in the transmission of several poultry pathogens, some of which are transmissible to humans. The zoonotic pathogen *S. enteritidis* is one of the most widespread zoonoses and causes nontyphoidal salmonellosis (Valiente Moro et al., 2007). This disease has the highest global human death rate, and is one of the most common diseases of food-borne origin and poultry products (Majowicz et al., 2010). *Salmonella* can survive internally in PRM for up to 4 mo and are transmitted to poultry by external cuticular contact, the ingestion of a blood meal, or when birds consume infected mites (Moro et al., 2007; De Luna et al., 2008). *Borrelia burgdorferi*, the cause of Lyme disease, and avian influenza A virus were recently added to the list of zoonotic pathogens potentially transmitted by PRM (George et al., 2015).

### Zoonotic Risks

Poultry red mite is a bird ectoparasite with low host specificity. As a result, mites can feed on mammals, including humans, when the natural host is not available. Human parasitosis, also called gamasoidosis, has been described in bitten humans (Cafiero et al., 2019). Erythematous papules and urticarial lesions in the skin are the usual clinical signs of gamasoidosis, and while these lesions can be distributed over the body, they are more frequently located on the arms, legs, and upper trunk (Pezzi et al., 2017). Regarding human gamasoidosis, 2 epidemiological scenarios (urban and occupational) have been described (Sioutas et al., 2021). Skin lesions in urban cases tend to be more severe than occupational lesions owing to extended exposure times, and their frequency has increased in recent years (Anderson and Meade, 2014). Although PRM is the most common cause of gamasoidosis, misdiagnoses often occur due to the difficulty of determining species, and recent investigations suggest that pigeon-specific *D. gallinae* L1 is more frequently involved in human gamasoidosis (Pezzi et al., 2017).

### Red Mites Are of Growing Concern in Human Diseases

Poultry red mite infestations induce human dermatological lesions, namely gamasoidosis, particularly in people living or working close to poultry, as described in Sections “Poultry Red Mite Infestation” and “Conventional Control by Inorganic Materials.” As the incidence of gamasoidosis has increased worldwide, closely related diseases are being undiagnosed. The severity of the disease caused by mites is worsened by the persistence of infestations, frequent treatment failures, and the potential transmission of zoonotic diseases, such as *Borrelia burgdorferi, Babesia*, and *Bartonella*, as described previously. For example, a 19% incidence of contact dermatitis was reported in a 2-yr survey of workers on 58 European poultry farms, even though many cases were misdiagnosed or unreported (Cafiero et al., 2011). Therefore, the actual incidence is higher than commonly assumed, indicating PRM infestation is a serious matter for the “One Health” initiative (Sigognault Flochlay et al., 2017). Both veterinary and human health implications of mite infestation are central focus areas of the European Cooperation in Science and Technology (**COST**) conference. In 2011, a group of European researchers claimed that red mites required inclusion as an occupational hazard for individuals working with poultry due to their role as zoonotic agents (Cafiero et al., 2011).

## CONVENTIONAL CONTROL BY INORGANIC MATERIALS

To keep PRM infestations under control and avoid the transmission of mites, much attention has been given to the conventional treatment of PRM using synthetic acaricides. However, the use of these chemical acaricides is limited because of safety regulations for human consumers (Sparagano et al., 2014). The levels of resistance in PRM has also increased, indicating a lower efficacy (Marangi et al., 2012). Numerous experimentally sustainable tools have been adopted for the control of PRM and are still being tried as alternative nonchemical treatments, such as plant-based products, plant-derived compounds, vaccines, and biological approaches (see the following sections). However, none of these seem to be sufficiently efficient for treating PRM (Decru et al., 2020). The only sustainable solution for controlling PRM infestations is to use multitactic integrated pest management programs (**IPM**), as suggested in previous reports (Arends and Robertson, 1986; Axtell and Arends, 1990; Harrington et al., 2011; Mul et al., 2015). However, in practice, this approach is still very limited in the poultry industry and is restricted (Mul et al., 2015; Tomley and Sparagano, 2018).

### Chemical Approach

Chemical treatment by organic compounds are commonly used to control the growth of PRM. Here, we briefly summarize synthetic pesticides and essential oils (Table 1).

**Table 1:** Examples of conventional poultry red mite control using chemical approaches.

Type	Tested substances	Mode of action
Selective synthetic pesticides	Organophosphate phoxim	Acaricidal
	Fluralaner-based product; Exzolt R	Acaricidal
Essential oils	*Azadirachta indica*	Acaricidal
	Neem oil formulation (RP03TM)	Acaricidal
	Carvacrol	Acaricidal
	*Artemisia sieberi* (Besser) oils	Repellent
	*Myrcia oblongata* (DC) oil	Repellent
	Transverbenol oils	Repellent
	Cinnamon bark and clove bud oils	Repellent
	Thyme oil	Repellent/acaricidal
	Lavender oil	Repellent/acaricidal
	Oregano oil	Repellent
	Garlic juice	Repellent

#### Pesticides

Acaricide selection is important to avoid the emergence of resistance in the pest, and to optimize the recommended dosages for successful treatment. Furthermore, side effects on nontarget species and the environment can be avoided and reduced. Unfortunately, no available synthetic chemical products are selective for PRM, indicating toxic effects on other insects and arachnids (Decru et al., 2020). The selection of synthetic acaricides is very difficult due to application restrictions, such as permission for use against PRM, and different regulations for products classified as biocides or veterinary medicines. The registrations of these products are dynamic and can change daily, making it necessary to regularly check and even require approval for their application during production, with some restrictions (Damalas and Eleftherohorinos, 2011). The organophosphate phoxim (ByeMite R) has been licensed as a veterinary medicine against ectoparasites for livestock, including layer hens. A previous study reported the high efficacy of ByeMite R in multiple systems after repeated application, but the emergence of resistance against phoxim has already been reported (Meyer-Kühling et al., 2007). Recently, a fluralaner-based product, Exzolt R, as a systemic acaricide against PRM, has been administered orally through drinking water; it can inhibit ligand-gated chloride channels in the nervous systems of mites, ticks, and insects (Gassel et al., 2014). Exzolt R killed mites very quickly (within 4 h under laboratory conditions), and 100% mite reduction occurred almost immediately in all tested units based on field studies (Brauneis et al., 2017; Thomas et al., 2017). However, the duration of efficacy varied greatly among the tested layer farms, ranging from 56 d to 238 d (Thomas et al., 2017).

#### Essential Oils

The effective use of mineral oils, diesel oil, petroleum, and plant oils (rapeseed and orange) for poultry red mite control has been illustrated in previous in vitro studies (Maurer et al., 2009b). These oils induce physical stigmata and prevent the normal breathing of mites (Decru et al., 2020). Nonetheless, because of the disadvantages of oils, such as staining the eggs, affecting the egg belt, and cleaning difficulties, only some oils have been used by farmers for years (Holt et al., 2011). Because mites will avoid oil spots, oils can be applied as barriers to successfully control mite infestations (Decru et al., 2020). However, these oils are currently not permitted for PRM treatment because of the lack of biocide registration. Lundh et al. showed that *Azadirachta indica* oil, known as neem oil, has a good acaricide effect associated with a low repellent effect at a concentration of 15 to 20% under laboratory conditions (Lundh et al., 2005). Camarda et al. tested its effectiveness under field conditions. After 3 nebulizations, the 20% neem oil formulation (RP03) caused a mite decline of 94.65 to 99.80% over 1 wk in poultry farms (Camarda et al., 2018). Other essential oils from the Lamiaceae family (Martinov), such as *Origanum* (L.), *Satureja* (L.), *Thymbra* (L.), and *Thymus* (L.), are also effective (Chorianopoulos et al., 2004). The major compound in these genera is a carvacrol, which is a good acaricide candidate with a toxic effect on PRM at concentrations above 1% (Kirimer et al., 1995; Tabari et al., 2015; Barimani et al., 2016). During in vitro contact bioassays, thyme and lavender oils had the highest repellent activity with, respectively, 80 and 40% of the oil-treated surface area being avoided by PRM (Nechita et al., 2015; Pritchard et al., 2016). Nevertheless, this type of experiment cannot be used to distinguish whether mites are repelled by olfactory or contact cues, and complementary studies with olfactometers are required (Deletre et al., 2016). Although *Artemisia sieberi* (Besser) oils, *Myrcia oblongata* oil, and transverbenol oils showed a significant repellent effect on PRM, the constituent compounds were not evaluated (Deletre et al., 2016; Santana et al., 2018). Lee et al. reported that when 2 essential oils (cinnamon bark and clove bud oils) and some of their constitutive compounds were tested, they exhibited repellent ability to PRM, except for cinnamyl acetate from cinnamon bark oil and 2 compounds from clove bud oil (eugenol and eugenol acetate) (Lee et al., 2019). A study by Camarda et al. investigated the formulation of 20% neem oil diluted from a 2,400 ppm azadirachtin concentrated stock against PRM, administered by nebulization 3 times per week (Camarda et al., 2018). The mite populations showed 94.6, 99.6, and 99.8% reductions after the first, second, and third application, respectively. The strong bioactivity of neem against PRM was highest 10 d after the first application, and its effects persisted for over 2 mo. Tabari et al. (2017) explored *Artemisia sieberi* essential oil against PRM, and the repellent activity was tested on adult mites over different time intervals, indicating that mortality was significantly higher in open containers than in closed containers, highlighting the key role of its highly volatile constituents (Tatami et al., 2001). Puvača et al. investigated the repellent effects of thyme (*Thymus vulgaris* L.), lavender (*Lavandula angustifolia* L.), and oregano (*Origanum vulgare* L.) essential oils on PRM in controlled laboratory conditions (Sasaki et al., 2005; Mkolo and Magano, 2007; Nechita et al., 2015). The best results were observed for lavender essential oil (96% mortality after 72 h) and thyme (82% mortality after 72 h) at a dose of 0.15 mg/cm^3^. After application to filter papers, the thyme (100% mortality at 72 h) and lavender (78% mortality after 72 h) essential oils showed significant persistent toxic effects for 15 and 30 d, respectively, exhibiting both toxic and repellent effects against PRM. Mohammadyar et al. showed that garlic juice was highly effective against poultry red mites (Ranjbar-bahadori et al., 2014).

### Biological Approach

**Figure 3:**
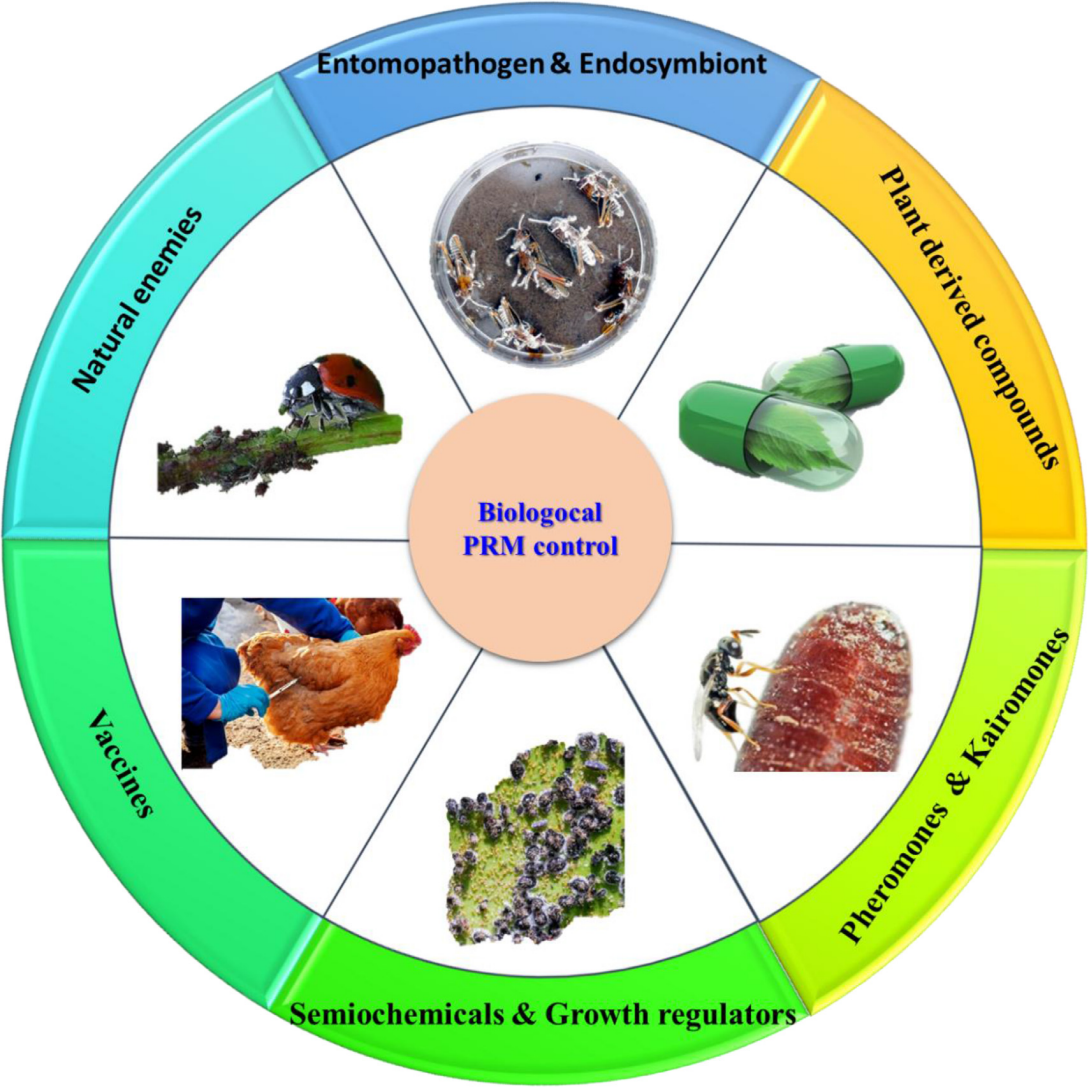
Schematic classification of biological approaches for poultry red mite (PRM) control.

Biological control is commonly adopted for food production. Spinosad (Elector R) has been reported as a biological acaricide that acts on the nervous system of mites and is registered for use in organic farming. However, actual field tests illustrated that the effect of Elector R were short lived, and it was insufficient as a stand-alone treatment. In this section, we consider biological organisms and biological substances, pheromones/kairomones, semiochemicals/growth regulators, and vaccines (Figure 3 and Table 2).

**Table 2:** Examples of conventional poultry red mite control using biological approaches.

Type	Tested substances	Mode of action
Natural enemies	*Androlaelaps casalis*	Prey on PRM
	*Hypoaspis aculeifer*	Prey on PRM
	*Hypoaspis miles*	Prey on PRM
	*Stratiolaelaps scimitus*	Prey on PRM
Entomopathogen and endosymbiont	*Beauveria bassiana*	Penetrate host
	*Metarhizium anisopliae*	Penetrate host
	*Trichoderma album*	Penetrate host
	*Cardinium* sp.	Acaricidal
	*Spiroplasma* sp.	Acaricidal
	*Nematodes*	
Plant-derived compounds	*Conocarpus erectus* extracts	Repellent
	*Cnidium officinale* extracts	Repellent
	*Portulaca oleracea* extracts	Acaricidal
	*Pistacia atlantica* extracts	Acaricidal
	*Thymus thyme* extracts	Acaricidal
	*Arctium burdock* extracts	Acaricidal
	*Tanacetum vulgare* extracts	Acaricidal
Pheromones and kairomones	Aggregation pheromones	Attractant
	Alarm pheromones	Attractant
	Immature female pheromones	Attractant
	Female sex pheromones	Attractant
Semiochemicals and growth regulators	Allomone	Attractant/repellent
	Triflumuron	Acaricidal
	Bm86	Boost immunity
	Dg-HRF-1	Boost immunity
Vaccines	Dg-CatD-1	Boost immunity
	Dg-CatL-1	Boost immunity

#### Natural Enemies

Naturally occurring enemies are promising candidates for controlling PRM, such as using ectoparasites that share living environments (Zriki et al., 2020). Some natural enemies are commercialized and can be confined to the release site by using controlled enclosed systems, where their effectiveness can be measured (Mul et al., 2015). *Androlaelaps casalis, Hypoaspis aculeifer, Hypoaspis miles*, and *Stratiolaelaps scimitus* (previously *H. miles*) naturally occur in layer houses and are genuine predators of PRM, although they are not specific predators of PRM, affecting their long-term adoption as a control strategy (Lesna et al., 2009; Roy et al., 2017). Nonetheless, natural communities of PRM enemies in the layer house can partly control poultry red mites. In enclosed systems, particularly in poultry houses, natural enemy dispersal is restricted (Wales et al., 2010). The field performance of natural enemies may be temperature dependent and limited by alternative pest control measures; however, many species have been reported in poultry houses (Chauve, 1998; Axtell, 1999). For example, the histerid beetle *Carcinops pumilio*, a bulky beetle that tends to dwell in manure, is often encouraged by poultry producers (Lesna et al., 2012). Therefore, predatory invertebrates show promise as part of a combination of treatments within the IPM strategy (Roy et al., 2017).

#### Entomopathogens and Endosymbionts

Entomopathogenic fungi, nematodes, and bacterial endosymbionts have potential as nonchemical controls against PRM; however, their use in poultry houses still has many issues and they are not yet commercially available (Tomer et al., 2018). The spores of fungi infect mites by adhering to the host's cuticle, germinating, penetrating, and then spreading into the host's body, which is specifically susceptible to *Beauveria bassiana, Metarhizium anisopliae*, and *Trichoderma album* under laboratory-scale conditions (Tavassoli et al., 2011; Immediato et al., 2015; Tomer et al., 2018). These studies showed a significant decrease in PRM numbers that persisted for 4 wk after treatment. However, some experiments in semicommercial conditions showed unsatisfactory control against PRM due to low humidity levels for fungal transmission and a lowered dose in poultry houses, while the effectiveness is highly dependent on the fungal strain if there is no selectivity for only PRM (Harrington et al., 2011; Immediato et al., 2015). Furthermore, these organisms may negatively affect the environment, leading to environmental disequilibrium (Pritchard et al., 2015). Nematodes have been used as mite control species, but they require specific environmental conditions, such as high humidity levels and free water, making them very difficult to use in poultry houses (Harrington et al., 2011; Sparagano et al., 2014).

Endosymbiotic bacteria coexist with many arthropods and are vital for host survival (Entrekin and Oliver, 1982). Endosymbionts are also vital for the control of PRM and their growth has been investigated. The removal of symbiosome bacteria (and yeasts), which contribute to this symbiosis, caused dramatic declines in host reproduction and growth, owing to their irreplaceable acquisition by vertical transfer only (Coba de la Peña et al., 2017). Endosymbiotic bacteria may exert detrimental effects on host reproductive potential by inducing cytoplasmic incompatibility and disrupting oogenesis (Xiao et al., 2021). Although some endosymbionts have been identified in PRM by DNA sequencing, additional studies are required before they can be applied as a treatment in practice (De Luna et al., 2009).

#### Plant-Derived Compounds

Plant-derived products with acaricidal and toxic activity as well as repellent or attractive effects have promising potential as alternative nonchemical methods against PRM (George et al., 2009b; Birkett et al., 2011; Gay et al., 2020). Plant-based products mostly have low toxicity to mammals, short environmental persistence, and low impacts on the environment, making them suitable for use in IPM strategies (Kim et al., 2007; George et al., 2008a, 2009b). In the earlier section we demonstrated that the efficacy of plant-derived essential oils against PRM was mainly due to their volatile components, which exhibited a neurotoxic effect rather than a mechanical one (Kim et al., 2004; López and Pascual-Villalobos, 2010; Na et al., 2011; Lee et al., 2019). Furthermore, the efficacy of plant-based products may be affected by environmental factors, such as humidity, dust, and other pesticides used (George et al., 2010; López and Pascual-Villalobos, 2010).

To date, plant species from at least 15 different botanical families have been tested, including *Commiphora holtziana* spp. *holtziana* (gum haggar), extracts of *Conocarpus erectus*, and methanolic extracts and fractions of *Cnidium officinale* that are repellent to PRM (Soulié et al., 2021). Although the repellent activity of these substances is expected to be relatively safe for nontarget organisms, the chemical compounds from the same plant species varied in quality and quantity between the batches studied (Thompson et al., 2003; George et al., 2009a; Ben Jemâa et al., 2012; Dardouri et al., 2019). Most studies have focused on the acaricidal properties of the plant extracts. Rajabpour et al. showed that aquatic and ethanolic extracts of *Conocarpus erectus* were repellents for PRM (Rajabpour et al., 2018). Kim et al. showed that the methanolic extract and fractions from the rhizome of *Cnidium officinale* exhibited 91.3% repellent activity on PRM. An additional study on (Z)-ligustilide, which is an isolated compound from *C. officinale* roots, showed an almost 100% repellent response using a T-tube olfactometer (Kim et al., 2018). Additionally, *Portulaca oleracea, Pistacia atlantica*, and *Terminalia chebula* Retz. extracts can be used as a botanical acaricide against *Oligonychus caffeae* (Roy et al., 2014; Rajabpour et al., 2019). Some plant-based acaricides are commercially available for use in layer houses in some countries. For example, MiteStop R (Alpha-Biocare GmbH, Neuss, Germany), which consists of a neem seed (*Azadirachta indica*) extract, has proven efficacy against PRM in vitro and in field conditions (Abdel-Ghaffar et al., 2008, 2009; Locher et al., 2010).

The repellent nature of plant products such as thyme (*Thymus* spp.), burdock (*Arctium* spp.), and tansy (*Tanacetum vulgare*) appear to be behind their effectiveness for controlling PRM in birds when used as a drinking water additive. The use of these in an integrated approach may be particularly effective for PRM control, which are more susceptible to acaricides (George et al., 2008b). However, the use of plant-based products in PRM control is associated with a relative lack of standardization and consequent inconsistent efficacy (Isman, 2006; George et al., 2009a; Mul et al., 2009).

#### Pheromones and Kairomones

Volatile organic compounds (**VOCs**) are an interesting alternative to classical acaricides for controlling mite populations, and several types of VOCs, such as pheromones and kairomones, have been identified and their properties have been studied (Vivaldo et al., 2017). Pheromones are molecules that influence the behavior of other individuals of the same species. Several pheromones, including aggregation pheromones, immature female pheromones, female sex pheromones, and alarm pheromones, are thought to attract mites (Sonenshine, 1985; Carr and Roe, 2016). To understand their effects, the identification and quantification of molecules within the pheromonal mix were analyzed, and additional tests were performed to characterize the behavior of mites when they were confronted with all or part of the identified molecules (Entrekin and Oliver, 1982; Steidle et al., 2014).

The aggregation pheromones of mites cause nonfeeding conspecific mites at different developmental stages to aggregate in a safe environment (Carr and Roe, 2016). Entrekin and Oliver studied 2 possible causes that might induce this behavior: thigmokinesis and the release of an aggregation pheromone (Entrekin and Oliver, 1982). Both stimuli affected mite clustering; however, chemical stimuli formed aggregates more efficiently. The aggregation phenomenon by PRM was evaluated by Koenraadt and Dicke; the attraction of 2 groups of conspecific mites showed that both fed and unfed mites were attracted by volatiles emitted by fed conspecifics (Koenraadt and Dicke, 2010a). This finding was consistent with a previous study by Entrekin and Oliver, where mite aggregation increased after feeding. Koenraadt and Dicke proposed that unfed mites were motivated to find a host to feed, while fed mites searched for a place to hide and reproduce after their blood meal (Koenraadt and Dicke, 2010a). It was concluded that the aggregation pheromone of PRM was composed of only 1 or several molecules, as observed in other mite species. For example, lardolure was the only aggregation pheromone identified in *Caloglyphus polyphyllae* and *Lardoglyphus konoi* mites (Kuwahara et al., 1994; Carr and Roe, 2016).

Alarm pheromones are emitted by an individual when stressed by an unsafe environment or injured (Carr and Roe, 2016). The detection of low concentrations of alarm pheromones can cause attraction and clustering behaviors in mites as survival strategies. Above a given concentration, the pheromone becomes repulsive, causing scattering and hiding. To date, the emission of alarm pheromones on behavior in PRM has not been studied; however, several compounds, such as neral and neryl formate, have been identified in other stigmatid mite species (Kuwahara, 2004, 2010). Mites (*Schwiebea elongata*) were attracted to neral when it was applied at low doses of 1 and 3 ng on filter paper; however, at higher doses (30 ng), the mites exhibited repulsive behavior (Maruno et al., 2012). Neryl formate has been identified in several species, including *Tyrophagus putrescentiae*, and leads to alarm behavior (Kuwahara, 2010; Skelton et al., 2010). However, high doses of neryl formate (10 and 100 ng) also attracted other species, such as *D. farinae* and *D. pteronyssinus*, indicating it can also act as an aggregation pheromone (Skelton et al., 2010). Therefore, it is essential to compare alarm pheromones that attract mites at low doses with aggregation pheromones that attract mites at all doses.

Two types of sex pheromones have been detected in mites: 1) immature female pheromones that only deutonymphs can emit, and 2) female sex pheromones (Sonenshine, 1985; Carr and Roe, 2016). During reproduction, male mites distinguish between deutonymphs and protonymphs by the immature pheromone signature emitted by deutonymph females, which, in *Tetranychus urticae*, is composed of citronellol, farnesol, and nerolidol (Regev and Cone, 1975, 1976, 1980). However, this immature female pheromone has not been identified in PRM, and normally only female sex pheromones are released to attract preferentially fed males to ensure high reproductive success (Sonenshine, 1985; Carr and Roe, 2016). Sometimes, the female sex pheromone may also be released by males, explaining the mounting behavior between males (Mori and Kuwahara, 2000; Mizoguchi et al., 2005). Although female sex pheromones have not been studied in PRM, different compounds identified in closely related species could be potential candidates. For example, 2-hydroxy-6-methylbenzaldehyde is used in different species, such as *Acarus immobilis, Aleuroglyphus ovatus, Cosmoglyphus hughesi*, and *D. farinae* (Kuwahara et al., 1992; Sato et al., 1993; Ryono et al., 2001; Tatami et al., 2001). The synergistic effects between sex pheromones and aggregation pheromones could also potentially be used to control PRM.

#### Semiochemicals and Growth Regulators

As host-related kairomones can act as attractants, a repellent allomone from ducks could also be an alternative to control PRM. This repulsive allomone derived from the uropygial gland of ducks has been synthesized and is commercially available. An important consideration in any attractant/repellent approach is the volatility of product, which is applied using a multitude of slow-release mechanisms (Maia and Moore, 2011). However, this property is unlikely to be used in attractants and/or repellents in PRM control.

Growth regulators either disrupt the formation of the building blocks of invertebrate exoskeletons (chitin), or interfere with maturation by mimicking or inhibiting juvenile hormone, leading to delayed or premature development of the pupae or adults, respectively (Brenner and Kramer, 2019). Growth regulators are commercially available and have been recently considered for use in controlling PRM. For example, triflumuron can reduce egg hatching by disrupting embryonic development as chitin inhibitor, indicating its potential use an efficient strategy in controlling mites when used in combination with acaricides (Chauve, 1998). Triflumuron is currently available for use in poultry and is marketed for general use against pests of domesticated animals housed in the United Kingdom.

#### Vaccines

Vaccines are an attractive alternative to acaricides; however, the development of vaccines against arthropods is very difficult because of the time required and the potential induction of new immune reaction responses in the host (Willadsen, 2004; McDevitt et al., 2006). The development of vaccines against PRM is hindered by a poor understanding of the mite-host relationship (Harrington et al., 2009b). In addition, there are few reports in the literature detailing the development of vaccines against PRM. Although the immunization of birds with somatic PRM antigens or homologous proteins from other mite species, such as *Dermatophagoides pteronyssinus*, has been attempted, no significant mortality in mites was measured (Arkle et al., 2008; Sparagano et al., 2014). Since advances have been made in genomics and transcriptomes of PRM were recently published, the development of PRM vaccines, including the search for candidate antigens, has gained momentum (Schicht et al., 2013; Burgess Stewart et al., 2018). Several studies have demonstrated the potential of both native (autogenous) and recombinant antigens for vaccination; however, autogenous vaccines and effective antigens (s) are not well defined (Wright et al., 2009; Bartley et al., 2015). In other words, the efficacy of autogenous vaccines has not been quantified, indicating that different results were obtained between batches.

Despite these difficulties, in a recent field evaluation of both an autogenous vaccine and a prototype recombinant vaccine, the autogenous vaccine led to a 78% reduction in the mite population, while the recombinant vaccine did not show any significant efficacy (Bartley et al., 2017). An alternative approach to the use of somatic mite proteins is the immunization of hens with recombinant proteins derived from ticks (Bm86) or mosquitoes (subolesin) (Harrington et al., 2009a). The in vitro mortality of PRM was reported to be 23 and 35% in the Bm86- and subolesin-immunized groups, respectively. A genomic approach was used to investigate a vaccine candidate against PRM using both *Dg*-HRF-1 (*D. gallinae* histamine release factor protein) and *Dg*-CatD-1/*Dg*-CatL-1 (recombinant cathepsin D-/L-like proteinases) (Bartley et al., 2012). In detail, the immunization of hens with *Dg*-HRF-1 yielded a significant 7% increase in PRM mortality using blood spiked with polyclonal IgY when tested in vitro (Bartley et al., 2009). The *Dg*-CatD-1 treatment groups were more efficient than *Dg*-CatL-1 groups, and mortality was significantly higher than that of the control groups 120 h after initial mite feeding. This study demonstrated the potential of developing a vaccine against PRM based on somatic or recombinant proteins. However, significant technical hurdles must be overcome before a commercially available vaccine can be developed and released.

## PHYSICAL CONTROL BY INORGANIC MATERIALS

Inorganic materials can be used to physically control PRM by dehydrating the membrane cuticle, which is derived from water and oil adsorption components. Furthermore, coupling several materials can enhance the desired physical control effect. During exposure to PRM, each inorganic material can be randomly distributed, adsorbed onto the surface of PRM, and formed into a localized compartment. Therefore, the inorganic material can interact with the environment and affect regulate water sorption in PRM, leading to physical damage. These specific properties of inorganic materials are a major advantage in controlling PRM. In addition, the sharp edge of each inorganic particle prohibits mites from accessing poultry by creating a stressful environment that prevents movement on or away from the habitant poultry site to another site. Detailed studies of these inorganic materials are discussed in the next section.

Inert dust, including diatomaceous earth (**DE**), kaolin clay, talc, and silica, are available inorganic materials that act as active biocidal substances for the treatment of PRM. Kaolin clay and talc are mineral materials that can be sourced from nature, while silica can exist in synthetic and natural forms, such as DE (Table 3). In several regions, only natural silica can be used on organic farms. Regarding the crystal structure of silica, the amorphous phase is considered relatively nontoxic, whereas the crystalline form is more harmful to the environment, animals, and human health (Merget et al., 2002; Schulz et al., 2014). However, the mode of action of silica against PRM is completely mechanical, and it acts by drying out the epicuticle of the exoskeleton of the mites, leading to the desiccation of the mite cell membrane due to the absorbent character of silicon dioxide (Kilpinen and Steenberg, 2009b). Furthermore, resistance to this physical reaction is less likely to develop than to single-target molecules (Schulz et al., 2014). Because dust consists of fine particles and can induce adverse effects on the respiratory tract of humans, the use of liquid silica-based products is an alternative to reduce these deleterious effects (Maurer et al., 2009b). However, some silica particles can become airborne when the fluid is dried and further dispersed by hens (Takeuchi et al., 2005). In vitro, DE seems more effective than synthetic silica, but field studies have shown that liquid silica seems to have a longer lasting effect than DE (Maurer et al., 2009b; Alves et al., 2020). Despite their effectiveness against PRM, physical control by inorganic materials is still at a very early stage due to several issues, such as shortages of inorganic materials and variability in the properties of the material, including absorption, chemical composition, particle size, and specific surface.

**Table 3:** Examples of conventional physical controls of poultry red mites using inorganic materials.

Type	Tested substances	Characteristic	Mod of physical effect
Natural	Diatomaceous earth (Insecto)	3 μm–1 mm in size	Sorptive/dehydrating
	Diatomaceous earth (Diamol)	3 μm–1 mm in size	Sorptive/dehydrating
	Diatomaceous earth (SilicoSec)	8–12 μm in size	Sorptive/dehydrating
	Diatomaceous earth (ProtectIt)	Modified with synthetic amorphous silica	Sorptive/dehydrating
	Diatomaceous earth (Fossil Shield 90.0)	0.5–31 μm in diameter	Sorptive/dehydrating
	Diatomaceous earth with plant	2% pyrethrum extract	Sorptive/dehydrating/repellent
	Kaolin clay	1–2 μm in size	Sorptive/dehydrating
	Talc	5–50 μm in size	Sorptive/dehydrating
Synthetic	Amorphous silica	24–167 m^2^/g in BET surface area	Sorptive/dehydrating/ovicidal
	Crystalline silica	Well-defined structure	Harmful to environmental, animal and human health

### Natural Inorganic Materials

Natural inorganic materials are widely used as a physical control strategy for PRM. Practically, these materials are commercially available and are allowed for use in actual field tests; however, these have only illustrated their effects for treatment-based effectiveness. To understand the mechanisms for their modes of action, each material should be classified and studied in detail in terms of their properties and physicochemical characterization. Here, we summarize and discuss the composition and properties of DE, kaolin clay, and talc.

#### Diatomaceous Earth

Diatomaceous earth differs depending on the blend of pure DE and other natural clays and minerals. In natural DE deposits, different amounts of silica were present depending on sedimentation conditions (Yazdimamaghani et al., 2018). Diatomaceous earth particle sizes range from more than 3 μm to less than 1 mm, but are typically 10 to 200 μm, and have low density and high porosity. The typical chemical composition of oven-dried DE is 80 to 90% silica, 2 to 4% alumina, and 0.5 to 2% iron oxide. Natural DE products that have been applied to poultry to date are Insecto (Natural Insecto Products, Costa Mesa, CA), Diamol (O.W.A. Kemi, Skanderborg, Denmark), SilicoSec (MiljøXuen, Gandrup, Denmark), and the DE products modified with synthetic amorphous silica ProtectIt (Hedley Technologies, Ontario, Canada), and Fossil Shield 90.0 (FS 90.0, Bein, Eiterfeld, Germany) (Kilpinen and Steenberg, 2009a).

**Figure 4:**
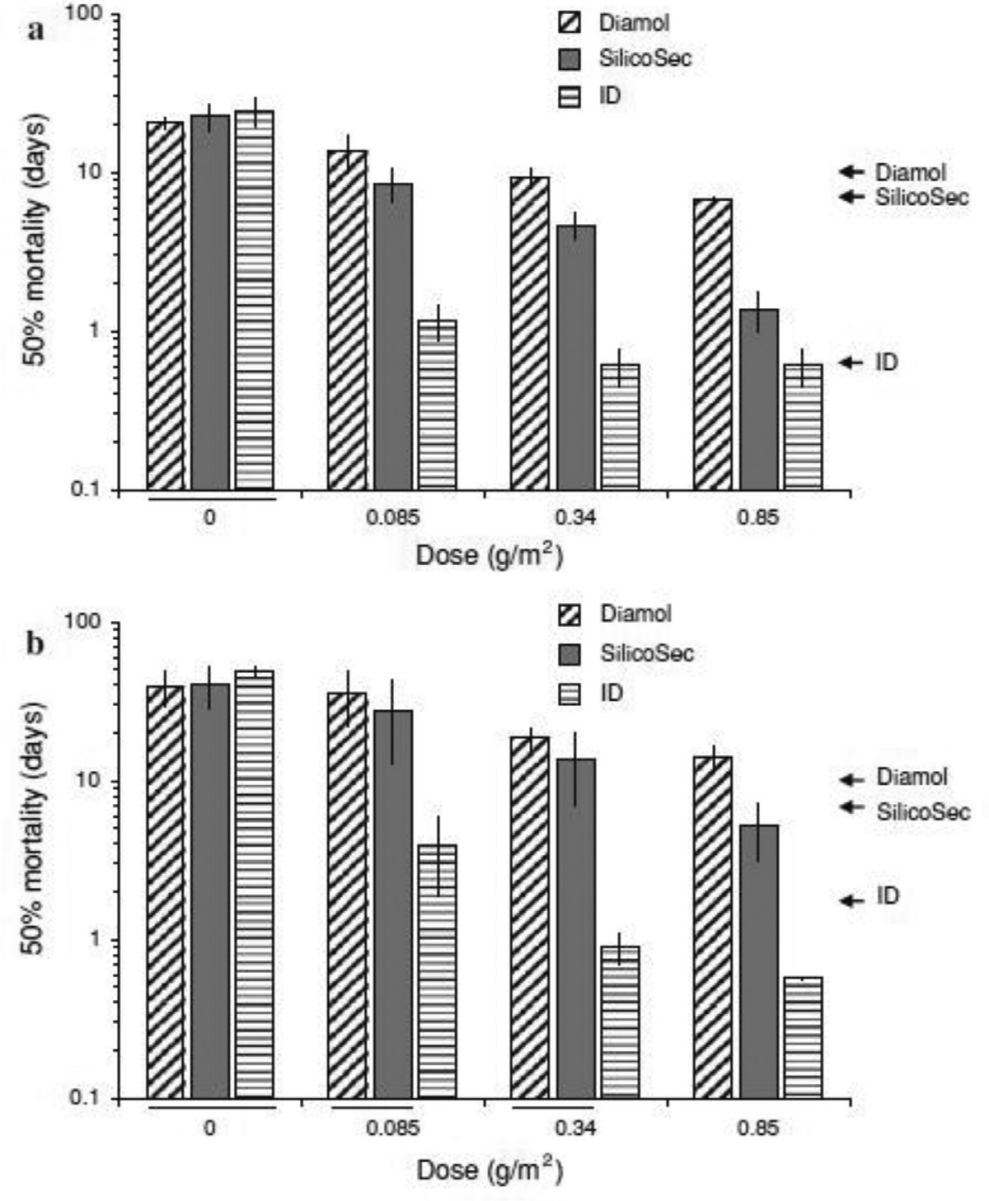
Results from the tarsal exposure trials with *Dermanyssus gallinae*. Average LT_50_ values (with standard deviations) for the 4 tests at each dose (and untreated control) at 75% RH (A) and 85% RH (B). Lines below the *x*-axis indicate results that are not significantly different (0.05 level of significance, Tukey-Kramer multiple comparison on log(*x* + 1) transformed data, SAS Institute 2000). Arrows to the right indicate LT_50_ values from the evaporation studies. There is a lower limit to the LT50 determination at around 0.6 d due to the 24 h observation interval from ref. Kilpinen and Steenberg (2009a,b). Copyright 2009, Springer Nature.

Kilpinen et al. designed 2 efficacy tests: evaporation and tarsal exposure. For the evaporation test, groups of 100 mites were collected in glass vials and placed in a closed box with a constant relative humidity (75 or 85%) and saturated solutions (NaCl or KCl) at 25°C (Kilpinen and Steenberg, 2009a). At variable intervals, the glass vials were weighed individually, the relative weight losses were calculated based on the initial weight of the mites, and the number of dead mites in each vial was estimated. In the tarsal exposure test, mites were exposed to different doses of DE on treated water-paper discs. After 24 h, the remaining mites on the treated paper were kept in plastic boxes with the same humidity conditions and observed daily for mortality over approximately 2 wk or until all mites were dead. In the evaporation experiment, data were obtained for both the weight change and mortality score. The mortality scores were fitted to a probit model, giving calculated values following the treatment until 50% of the mites were dead (mean lethal time or LT_50_). Data from the tarsal exposure tests were analyzed by calculating the LT_50_ for each experimental condition. These analyses proved that most DE doses cause almost complete water loss within 24 h at 75% relative humidity (**RH**) and 25°C, including the maximum dose of DE with a large amount of water. This indicates that mites cannot compensate for water loss by consuming a new blood meal. Additional water loss in DE-treated mites is due to evaporation across the mite cuticle (Ebeling, 1971). However, in the described experiment, the weight changes were no longer detectable in faster-acting treatments at the transition point where the mites started dying. In general, modified DE had higher efficacy than pure DE. Pure DE was as efficacious as ProtectIt when LT_50_ values were compared, and SilicoSec killed mites significantly faster than pure DE and Diamol, as shown in Figure 4. This suggests that inert dust containing a high proportion of silicon dioxide may have pesticidal effects. For the most efficient product tested, exposing the mites to treated surfaces for 24 h confirmed that mites were able to pick up enough product to be killed within less than a day. The doses applied in this study were generally much lower than those recommended by the companies. For example, the recommended application rate of Fossil Shield is 1 to 3 g per layer hen in cage systems, which is notably higher than that under in vitro conditions. This suggests that the mites were susceptible to low doses of inert dust, indicating that the treated surfaces were covered by environmental dust and debris. For Insecto and Diamol, mite weight losses of approximately 20% were recorded (18.2 and 21.1%, respectively). Slightly higher values were obtained for ProtectIt (25.9%) and SilicoSec (27.9%). Finally, FS 90.0 resulted in the highest weight loss of 37.3%. The LT_50_ data showed that high weight losses resulted in low LT_50_ values and vice versa. An important factor to consider was the humidity, where the general theory for the mode of action of inert dust to destroy the protective wax layer of the cuticle is prolonged depending on ambient humidity conditions. However, there was no obvious relationship between DE effectiveness and small changes in the humidity.

Mullens et al. used beak-trimmed, caged laying hens (25–45 wk of age) that were initially infested with 20 to 30 adult northern fowl mites (**NFM**) for 3 to 5 wk before treatment. Then, DE treatments were prepared by dispersing 60 g of DE in either 1) 440 mL of deionized water, 2) sulfur diluted with deionized water (15.6% sulfur in Trial 1 and 5.3% in Trial 2), or 3) a low concentration of sulfur. Hens were treated with these solutions using a handheld spray bottle or a 6-L pump pressure (garden) sprayer to ensure that the material remained in suspension. The DE formulation used was SilicoSec, which contains 92% SiO_2_ with average particle size of 8 to 12 μm (Athanassiou et al., 2005). Temperature plays an important role in determining the efficacy of a given DE formulation against mites. Generally, temperature increases mobility, resulting in more DE particles being attached to the cuticle (Fields and Korunic, 2000). With this DE formulation, 2 consecutive weekly treatments were required to achieve a DE level that controlled the mites (wk 10 of trial 2). Diatomaceous earth can be effective for suppression of PRM when applied thoroughly to its hiding places (cracks and crevices), and the same has been shown for NFM infesting human dwellings (Kilpinen and Steenberg, 2009a; Maurer et al., 2009a). Diatomaceous earth liquid formulations can be easily applied to hens by hand, but further testing is required to evaluate its use on a larger scale. Diatomaceous earth slurries can easily clog or damage many typical pesticide sprayers, sulfur can irritate the eyes and lungs, and DE dusts have inhalation risks.

Maurer et al. experimented with 3 different liquid formulations of DE (pure DE, D1; DE with essential oils, D2; and DE with pyrethrum, D3) in vitro. Five adult female mites were transferred into plastic vials treated with the 3 liquid test products, and mite survival was observed after 4, 24, and 168 h. In farm experiments, the mites were sampled for 4 d prior to treatment administration and during the next 3 and 4 d after treatment. Treatments D1 and D3 caused rapid mortality of the mites, while D2 was significantly less effective in vitro but still more effective than the control. The difference between D2 and the other treatments was that different qualities of DE were used, indicating that the quality of the raw material was the dominant factor influencing the effectiveness of DE products. This effect of DE was relatively small, irrespective of the mode of application. Practical experience has shown that good mite control can be achieved with repeated treatments. The different results between the farm and in vitro studies require further investigation.

Alves et al. prepared and evaluated the acaricidal activity of a liquid DE formulation in the laboratory and its association with mechanical cleaning in the field (Alves et al., 2020). A 10% DE liquid preparation was administered for treatment associated with mechanical cleaning. Forty-two days after the first DE treatment, a population reduction of 94.7% was observed compared with the initial population of mites. These results confirm that DE applications in combination with mechanical cleaning can be an alternative for effectively controlling PRM, which can also contribute to avoiding mite resistance from using chemical acaricides. It was observed that the effect of the DE formulation declined after repeated treatments, which could be related to flock age or the accumulation of formula in laying hen houses over time, hampering its efficacy (Mul et al., 2017). This problem can be overcome by mechanically cleaning surfaces prior to DE application, which has been proven to increase the efficacy of its acaricidal effects.

#### Kaolin Clay

Rocks rich in kaolinite minerals and kaolinite are called kaolin or china clay, which is a white soft clay that is an essential ingredient in the paper, rubber, and paint industries (Liu et al., 2016). Kaolinite is composed of Al_2_Si_2_O_5_(OH)_4_ and a layered silicate mineral structure, with 1 tetrahedral sheet of silica (**SiO_4_**) linked through oxygen atoms to 1 octahedral sheet of alumina (**AlO_6_**) (Mohsen and El-maghraby, 2010). Kaolin is a porous inert compound that is widely used as a carrier or diluent in dry formulations. The anticipated limited effect of chemical and microbial pesticides on mites indicate that kaolin might be a useful alternative for controlling mites (Subramanyam and Roesli, 2000).

Kilpinen et al. compared the effectiveness of the kaolin through the slowest-acting treatments using the exponential evaporation model. The kaolin treatments killed mites faster, providing a good approximation of mite weight changes over the experimental period. The weight loss after 24 h and LT_50_ values varied significantly for the different types of treatments at both RH levels. At the lowest humidity, kaolin caused slightly higher weight loss (11.9%) after 24 h compared with the control group (9.5%). At 85% RH, the weight losses were smaller and the LT_50_ values were higher. Based on the LT_50_ values and weight change data, the LT_50_ was lower and the weight change was higher at 75% RH than at 85% RH. The kaolin treatment had no observable effects on egg production.

Tests by Mullens et al. showed that kaolin clay was considerably more effective than the tested DE formulation (Mullens et al., 2012). The kaolin clay treatments were suspended in solutions of 60 g of clay in 440 mL of deionized water or 5.3% sulfur diluted with deionized water; the sulfur treatment was applied at wk 3 only. Hens were scored weekly for 12 wk and again at 14 wk. Kaolin clay was considerably more effective for controlling mites. Single applications of kaolin significantly reduced NFM for 1 to 2 wk, and 2 consecutive weekly applications controlled them for 2 to 3 wk. Kaolin clay is frequently used for plant crop pests in organic situations.

#### Talc

Talc, or talcum, is a clay mineral composed of hydrated magnesium silicate with the chemical formula Mg_3_Si_4_O_10_(OH)_2_. This mineral is used as a thickening agent and lubricant. In some experiments on mites, kaolin and talc were included for comparison because of their porous structure and function. Few studies have been conducted and discussed thus far.

Kilpinen et al. compared the slowest-acting talc treatments. Approximate mite weight change data fitted well during the experimental period until the mites began to die. The talc caused slightly higher weight loss (12.1%) than the control group (a 25% increase compared with the control). There were significant effects of humidity on both the LT_50_ values and weight change data; the LT_50_ was lower and the weight change was higher at 75% RH than at 85% RH. However, similar to kaolin, talc had no effect on egg production.

### Synthetic Inorganic Materials

Synthetic inorganic materials can be physical alternatives for controlling PRM. Practically, these products are commercially available and have various structures, surface areas, and pore sizes/volumes, which can affect their interaction with PRM. To help understand their control mechanisms, the physical properties of each type of material are discussed and compared. In particular, the water or oil adsorption properties are important factors in the physical treatment of PRM. Herein, we summarize and discuss several synthetic silica materials to evaluate their effectiveness.

#### Synthetic Silica

Silica, known as silicon dioxide (**SiO_2_**), is commonly found in nature as quartz and in various living organisms (Martin, 2007). Silica is one of the most complex and abundant materials and exists as a compound in several minerals or synthetic products. As previously discussed, natural and synthetic silica-based products are a biocidal option for controlling PRM. Typically, synthetic silica can be prepared in an amorphous or crystalline phase. Crystalline silica is structurally well-defined, while amorphous silica lacks long-range order, and their structures are strongly dependent on kinetic and environmental factors owing to a flat energy landscape (Trofymluk et al., 2005). Silicon dioxide has water/oil adsorption properties, which cause dehydration of the cell membrane of PRM after absorbing lipids from their cuticle (Schulz et al., 2014). One of the advantages of silica products is their low oral toxicity (Yoo et al., 2022).

Kilpinen et al. tested 2 pure synthetic amorphous silica products: Rentokil (Glostrup, Denmark) and a confidential formulation under testing (ID) (Kilpinen and Steenberg, 2009a). The more efficient product was ID, which caused 50% mite mortality after 0.6 d at 75% RH and 1.7 d at 85%. While these results showed the efficacy of ID, the observation intervals were too long to precisely determine the LT_50_. Nonetheless, synthetic silicon dioxide is less dependent on lower humidity because of the hydrophobicity of these products, while it was susceptible to an increase from 75% RH to 85% RH.

Maurer et al. tested one synthetic amorphous silica on adult female mites by transferring these into the plastic vials with 0.05 or 0.005 g (9 or 0.9 mg/cm^2^) of powder or a liquid formulation containing 0.05 g of dry silica (Maurer et al., 2009a). The mites were kept at 27°C in dark, and surviving mites were counted after 4, 24, and 168 h under a binocular microscope during the experiments. Female mites did not produce eggs after the silica powder treatment, indicating that the synthetic amorphous silica powder was ineffective. Synthetic amorphous silica is commonly used as a desiccating agent for stored-product pests (Collins, 2006). The liquid formulations of synthetic amorphous silica completely suppressed the reproduction of PRM females and had a longer residual effect in the in vitro experiment. Field experiments in infested layer houses revealed a longer residual effect of liquid amorphous silica. The different results obtained on farms and in vitro studies were investigated. These indicated that the good physical effects of silica against PRM could be enhanced by the selection of high-quality raw materials and by means of the formulation. However, the physicochemical characteristics of silica were not tested, which can distinguish the effects of high quality. In other words, synthetic amorphous silica was tested without understanding its basic properties, which can affect the characterization of the materials tested.

**Figure 5:**
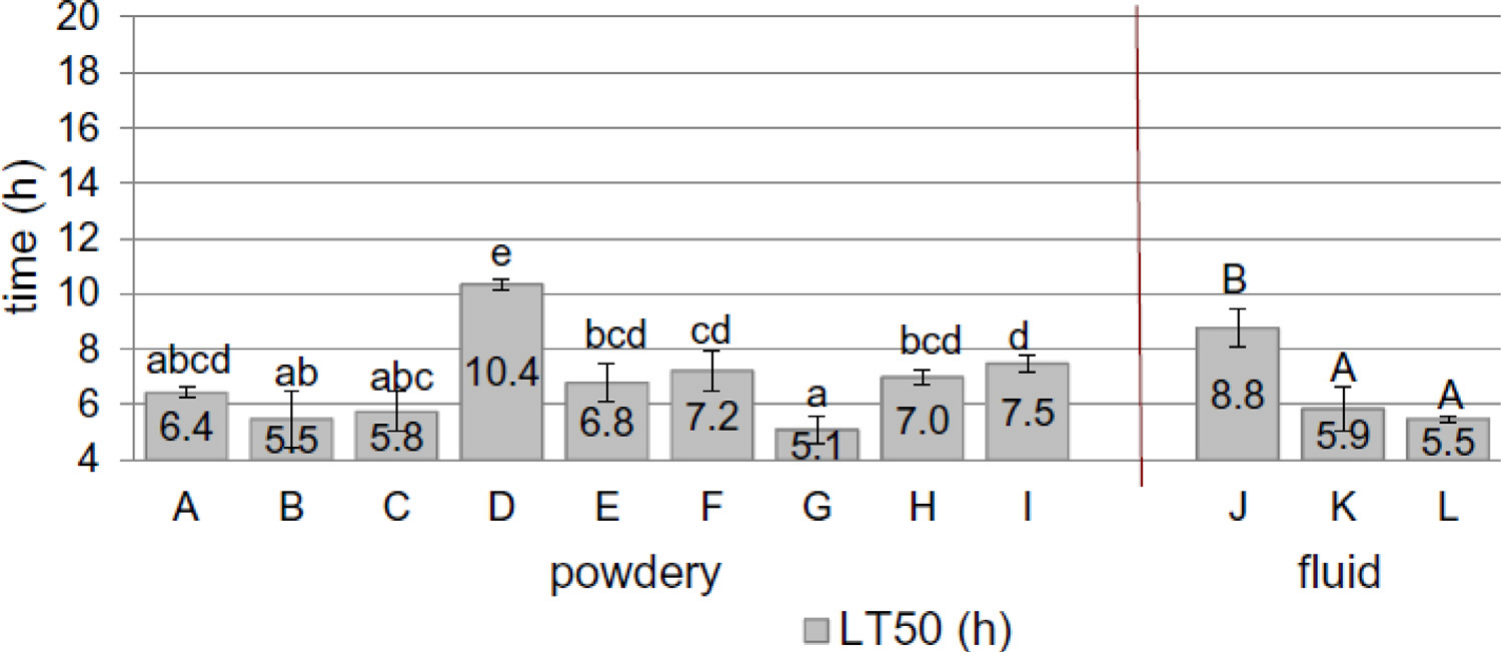
Efficacy of silicon dioxide-based products available on the marked (A–L), differentiated according to their method of application in the field (powdery or fluid) on the field mite strain (Tukey's HSD test (*P* < 0.05), different letters indicate significant differences) from ref. Schulz et al. (2014). Copyright 2014, Springer Nature.

**Figure 6:**
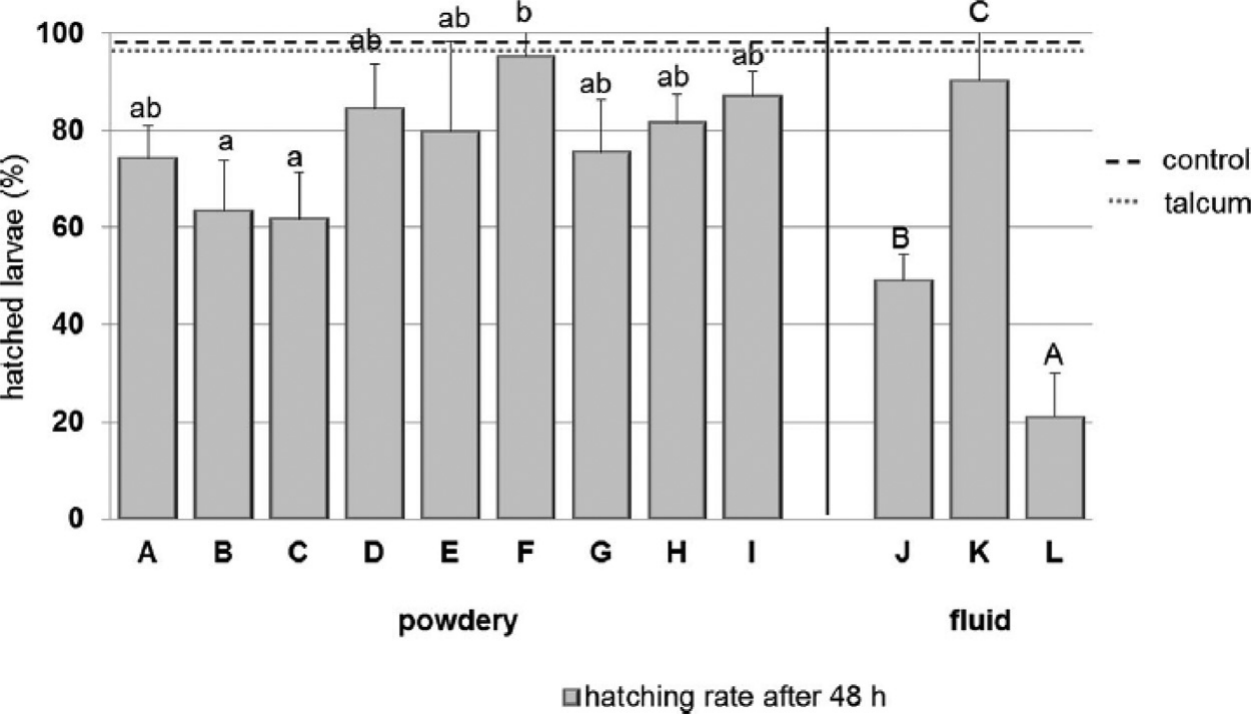
Ovicidal efficacy of silicon dioxide-based products available on the marked (A–L), differentiated according to their purpose of application in the field (powdery or fluid); on eggs of the laboratory mite strain (Tukey's HSD test (*P* < 0.05), different letters indicate significant differences); horizontal broken lines show the hatching rate of untreated eggs (control 98%) and eggs treated with talcum (talcum 96%). from ref. Schulz et al. (2014). Copyright 2014, Springer Nature.

Ulrichs et al. tested 12 silica-based synthetic amorphous silica formulations. However, they did not discuss the detailed contents of each sample. For ease of application, some silica fluid and powder formulations were tested, although the mode of action was equal to the desiccation properties of the formulation. In contrast to other studies, they first evaluated the tested materials themselves. To investigate the reactivity of silica, its surface area and morphology, including size, shape, and state of aggregation, were characterized in advance. Scanning electron microscopy revealed differences among the materials, and important characteristics that can affect the interaction between the silica and mites were investigated. X-ray fluorescence was used to analyze the SiO_2_ content, specific surface area (BET surface), cation exchange capacity (**CEC**), and water absorption capacity (**WAC**). The photometrical Cu-triene method was used to measure CEC (Meier and Kahr, 1999). The WAC test was performed by drying 1 g of each sample in aluminum cups (4 cm Ø). The absorption capacity was measured by the percent weight change (wt%) after 48 h at 50% RH compared with the weight of samples dried in the oven at 105°C. For the laboratory and field tests, PRM were collected from a conventional backyard hen house using cardboard traps (5 × 5 cm) taped underneath the perches. At midnight, the mites were transferred to the laboratory and treated with different silica samples. The acaricidal efficacy was determined for each product by comparing the LT_50_ values calculated from the mortality rates of 3 replicates. To determine ovicidal activity, mite eggs from the laboratory strain were treated directly with silica. In detail, 3 petri dishes were loaded with approximately 25 fed adult mites each and kept in a climate chamber at 25°C and 70% RH for 48 h. The results of the comparison of the silica efficacy against laboratory and field mite strains are shown in Figure 5. Acaricidal efficacy was observed for all tested silica products, with significant differences in the speed of action (LT_50_) values. In both experiments, the ranges of the LT_50_ values overlapped for all fluid- and powder-type products. However, there were significant differences in the ovicidal activity of the powder products, and the hatching rate was above 60% (Figure 6). The fluid products showed significant ovicidal activity. For example, the synthetic silica-treated fluid formulation caused the lowest hatching rate (21%), whereas the highest hatching rate (95%) was in the synthetic powder treatment. According to the electron microscopy analysis, the powder and fluid products resulted in clear acaricidal activity against the laboratory mite strain, but their effects differed among samples.

Other studies on the relationship between efficacy and morphology or size of silicas have mainly been conducted with insects. Many studies have investigated the acaricidal efficacy of silica-based products, but information on the parameters that determine their efficacy against blood-sucking acari is rare (Mewis and Ulrichs, 2001; Kilpinen and Steenberg, 2009a; Maurer et al., 2009a). Generally, the toxicity of amorphous silica depends on its intrinsic toxicity, surface chemistry (which makes contact with the cell), and morphology (size, shape, and state of aggregation) (Zhang et al., 2012). Ulrichs et al. tested the efficacy of fluid and dry formulations of silica against PRM and examined their biophysical properties.

The laboratory analysis and electron microscopy showed many differences between the tested products with respect to parameters such as the SiO_2_ content, BET, WAC 50, CEC, and LT_50_ values. In contrast to the effect of the SiO_2_ content on the speed of action, the BET, CEC, and WAC 50. The actual results significantly correlated with the model predictions. The BET and WAC were positively correlated, reflecting the lipid absorption capacity, which is partly responsible for their contribution to the efficacy of the product (Badii et al., 2014). In previous studies, silica was tested on different insect species to investigate if differences in the insects’ surface-to-volume ratio, water absorption ability, and cuticular compounds affected silica efficacy (Mewis and Ulrichs, 2001). In addition, types of formulations (powder and fluid) were compared. While fluid formulations of silica products are easy to apply, these were found to be ineffective against PRM (Maurer et al. from the Organic congress meeting). All silica treatments showed a strong acaricidal effect against PRM. However, the acaricidal efficacy significantly differed between products. Fluid silica instantly killed mites upon contact. Mites were possibly drowning in the fluid droplets applied onto surfaces, and an additional acaricidal effect would be possible using this matrix. The fluid formulation of synthetic silica was the most effective among the tested fluid samples. When other acaricides or additives were added to the silica, the acaricidal effect can be improved, which is well known in practice. Nonetheless, the similar LT_50_ values between fluids and dry powders are possibly misrepresentative of what occurs in situ; dry powders can reach much smaller cracks and crevices where mites hide (Abdel-Ghaffar et al., 2008). Therefore, the actual LT_50_ value in situ would be much lower for fluids than for dry silica products. In addition to using standardized climate conditions during the experiment, the products were adapted to the temperature and humidity prior to the experiment. The preadaptation of silica to the surrounding humidity is crucial for the correct comparison of the silica efficacy rates and errors. High RH causes these substances to lose their efficacy quickly.

Blood meal conditions also differ between the field and laboratory tests. The feed content in mites can play an important role in the efficacy of silica because its mode of action is desiccation, and many organisms can remetabolize water from food (Akbar et al., 2004). Different authors observed that insects can metabolically gain water from feed and it is believed that PRM obtain almost 90% of their water from blood (Beugnet et al., 1997).

Several reports have discussed the relationship between insecticidal efficacy and the morphological parameters of silica particles (Faulde et al., 2006; Mucha-Pelzer et al., 2008; Islam et al., 2010). It has been extrapolated that high BET surface values result in a faster ability to absorb lipids from insect epicuticles (Faulde et al., 2006). Practically, the results showed that higher specific surfaces increased the mortality rate of blood-sucking acari. However, the WAC 50 had a negative impact due to the water saturation of the silica, which reduced its absorption capacity for lipids from the cuticle (Ulrichs et al., 2008).

The above findings demonstrated that the mode of action for the acaricidal effect of silica was physical. In addition to determining the speed of action, it is important to determine how long the material is active against mites. For this purpose, the ovicidal effect of all materials on the mite population was investigated. For egg mortality, silica with the lowest WAC and BET killed more eggs than those with the highest WAC and BET values. In addition, the water saturation of products induced by the environment reduced their capacity to absorb lipids. Even though the ovicidal effect was not very high, freshly hatched larvae died within hours after treatment with the silicas. Therefore, larvae seem to be more sensitive than eggs to silica treatment. Differences in efficacy of fluid formulations of silica were difficult to determine. For example, the highest WAC resulted in the highest egg mortality, whereas the medium WAC value showed the poorest performance against eggs. Therefore, the WAC value was not related to silica efficacy. The BET value was not related to the ovicidal effect, and the specific surface area of the 2 products was similar to that of the eggs. A previous report discussed that smaller particles could penetrate the lungs of humans and animals more deeply, inducing new problems (Limbach et al., 2005). In addition, smaller-sized particles are generally more difficult to apply because of difficulties in scaling up production. Based on these results, the material should be carefully selected due to vast differences in the product efficacy rates. Furthermore, the advantages and disadvantages of both fluids and powders need consideration when selecting which product to use, including application difficulty, and ensuring the material can be applied to mite hiding places to ensure the products make contact with the mites.

## FUTURE PERSPECTIVE FOR INORGANIC MATERIALS

### Sorption and Abrasion Mechanism for the Removal of the Mite Epicuticle

The purpose of this section is to discuss the epicuticular lipid as a water barrier, explain the physical chemistry of its removal by means of inorganic materials, and discuss current uses and outlook for this mechanism in mite control. For pest control, inert dust may damage the pest cuticle through abrasion, absorption, and the release of subcuticular compounds. The combination of the increased availability of water and other nutrients for the removal or mitigation of inhibitory materials can lead to altered adhesive properties and physical disruption of the epicuticular lipid layers, causing excessive water loss due to desiccation (Zeni et al., 2021).

In the mite cuticle, a thin lipoprotein cuticulin covers the chitin protein procuticle. The surface of cuticulin is known as the epicuticle, which is initially hydrophobic but then becomes hydrophilic when tanned by quinones originating, at least in part, from the oxidation of “polyphenols” (Wigglesworth, 1947). The epicuticle is generally a solid wax, and the lipids consist of hydrocarbons, wax acids, esters, alcohols, diols, aldehydes, phospholipids, and other minor constituents (Chibnall et al., 1934). A thin layer of lipid resting on the cuticulin provides the principal water barrier of the epicuticle, which would be readily removed by adsorption due to the influence of physical stress on the surface (Wigglesworth, 1985; Wang et al., 2016). Usually lipid replacement is facilitated by water loss to prevent excessive desiccation, with the rapid replacement of wax molecules after the removal of principal water barrier on the cuticulin (Gibbs, 2011). The penetration of aqueous solutions through insect wax increases with increasing pH because of the saponification of the acids and esters following the hydrolysis of ester linkages (Aslan et al., 2015).

**Figure 7:**
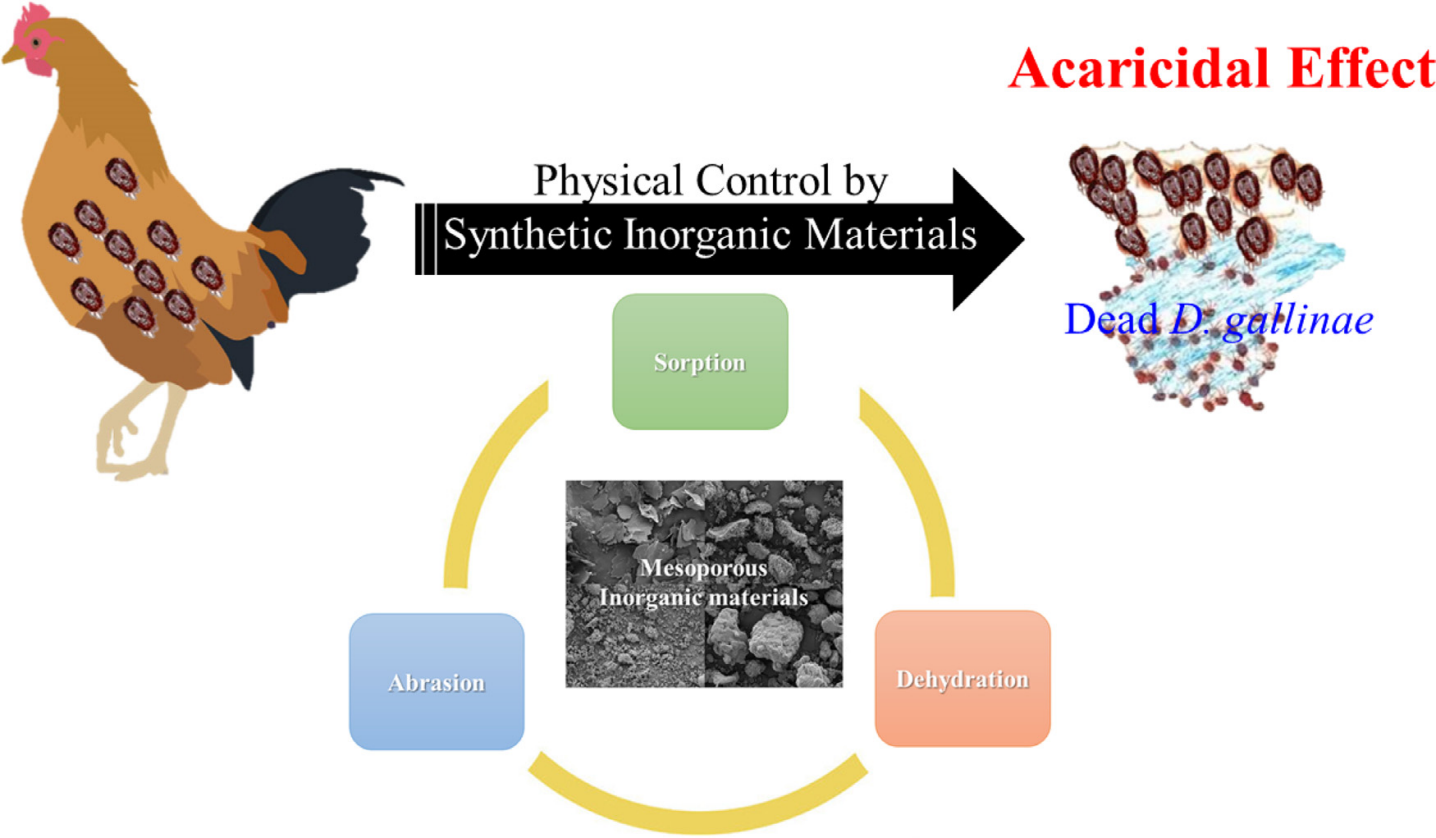
Schematic illustration of future perspectives for alternative inorganic materials as physical mechanisms for poultry red mite (PRM) control and a synergetic strategy.

Inorganic fine powders cause abrasion in most insects by removing the epicuticular wax to the extent that desiccation occurs, and some investigators found that the insecticidal efficacy of dusts was enhanced by increasing the hardness and abrasiveness of the particles (Ranabhat and Wang, 2020). Depending on the insect species, adsorbent dust was not as effective as abrasive dust, even though some adsorbent dust killed pests rapidly (Khayan et al., 2019). Nonetheless, adsorbents, such as alumina and colloidal silica, are highly effective. Kitchener et al. proposed that the epicutide fat film may be preferentially attracted to the surface of a solid particle and adheres and orientates itself on the surface of the cuticle without a defined crystal structure (Khayan et al., 2019). The fat film spreads over the particle by surface migration, and the hard wax occurring on the epicuticle of most insect species can be absorbed; this absorption depends entirely on the physical properties of the particle (Ebeling, 1971). Therefore, 2 different mechanisms operate for the insecticidal effects of inorganic materials. First, sorptive powder or liquid formulations, such as silica aerogels, activated charcoal, commercial bleaching earths, and acid-activated clays, remove the epicuticular wax based on the superiority of the sorption mechanism by disrupting the epicuticular lipid, leading to dehydration of the mite membrane, as discussed above in detail. In addition, insecticidal efficacy is closely correlated with specific surfaces, with large pores admitting wax molecules. Second, the insecticidal efficacy of nonsorptive dusts depends largely on their abrasiveness (Parkin, 1944). Therefore, the mode of action of inorganic materials is derived from their physical properties, which indicates that the most effective materials use abrasion, adsorption, and dehydration mechanisms, as described in Figure 7 (Cotton and Frankenfeld, 1949).

### Alternative Inorganic Material Candidates and Synergetic Strategies

As discussed above, it can be concluded that the adherence of inorganic materials to a red mite's body induces the abrasion or absorption of cuticular waxes, disrupting the membrane. Synthetic materials are alternative acaricide candidates that have important properties such as high-water adsorption capabilities and causing surface abrasion. In this section, some synthetic mineral materials (aside from synthetic silica) that are utilized as food additives are proposed as candidates for PRM control for their acaricidal effects. Porous materials with developed pore structures and large specific surface areas are vital for achieving high adsorption (Wang et al., 2020).

#### Phosphate-Based Minerals

Phosphate-based minerals, such as tricalcium phosphate, are used as supplemental mineral additives in processed foods and flour, and several studies have shown their toxic effects against stored-product insects and mites (Carrodeguas and De Aza, 2011). The particles attached to the mite body formed a thin layer that was toxic to the mites, and dead mites were observed with stretched bodies and legs after interacting with tricalcium phosphate. This mode of action is a physical effect on the surface of the mite, similar to that of other inorganic materials. The high doses required to produce acaricidal effects, however, prevent its application due to potential human health problems as inhalation toxicity tests have not been investigated in detail. Nonetheless, tricalcium phosphate is a biocompatible and eco-friendly material that is widely used in biomedical applications, such as self-setting osteotransductive bone cements and biodegradable bone repair, and its chemical synthesis is easy and inexpensive (Carrodeguas and De Aza, 2011).

#### Silicate-Based Minerals

Silicate-based minerals materials, such as calcium silicate hydrates (**C–S–H**), are good candidates due to man-made synthesis of the materials, which means the morphology and size distribution can be controlled. Water molecules invading the silicate chain are affected by the hydrophilicity and hydrophobicity of the silicate surface (Giovambattista et al., 2009). A previous study quantified the water distribution in C–S–H samples and determined the relative proportions of the water absorbed on the external and internal surfaces (Roosz et al., 2016). Based on the water vapor isotherms, C–S–H had external surfaces displaying multilayer adsorption of water molecules owing to its hydrophilic surfaces, and interlayer water also displayed irreversible swelling/shrinkage behavior. From this quantification of the water distribution, it was determined that the adsorption characteristics of the water molecules on C–S–H could be influenced by the structure of C–S–H. Generally, the calcium-silicon (**C/S**) ratio range was 0.67 to 1.67, which had a great influence on the adsorption properties of C–S–H (Hou et al., 2018). When the C/S ratio was >1, the maximum number of adsorbed water molecules increased with an increasing C/S ratio, indicating that the water adsorption property can be controlled by the synthesis method. In addition, the amorphous phase and rough irregular morphological shape of C–S–H induce abrasion without causing toxicity in humans and hens. Furthermore, the postmilling process helped reduce the particle size and produced rougher and sharper morphological surfaces to enhance the abrasive effect on red mites. In addition, as calcium silicate is a good delivery vehicle for drug loading, plant-derived compounds or hormones could potentially be loaded into its pores to provide synergetic acaricidal effects for red mite control.

#### Carbonate-Based Mineral

Carbonate-based mineral materials, such as mesoporous magnesium carbonate (**MMC**), are efficient moisture absorbers owing to their high affinity for water and large surface area (Pochard et al., 2014). Magnesium carbonate is widely used as a food additive in food processing applications as well as a source of magnesium in dietary supplements. Mesoporous magnesium carbonate can take up oil, even if it has already taken up as much water as silica. For example, the water adsorption capacity of silica preloaded with 50 wt% oil was reduced by more than 50%, but MMC maintained approximately 80% of its moisture absorption capacity when loaded with 50 wt% oil (Bamford, 2021). When water molecules are adsorbed, they readily interact with both the MMC surface and its bulk structure owing to their enhanced mobility, thereby creating a dual absorption effect. As discussed earlier, combining plant extracts with DE effectively controlled red mites; MMC with adsorbed essential oil would produce a synergistic effect against mites through the release of repellent oils and physical effects on the surface membrane of the mites. Recently, MMC has been subjected to a toxicological evaluation through in vitro cytotoxicity, skin irritation, and acute systemic toxicity tests in vivo. These tests showed that the material was nontoxic to human dermal fibroblast cells and had an antibacterial effect against the gram-positive bacterium *Staphylococcus epidermidis* (Welch et al., 2016). It has a broad particle size range with a rounded, slightly irregular and needle-like shape, and rough surface structure, indicating that different morphological shapes and diameters can be controlled by synthesis conditions, such as the reaction temperature, initial concentration, stirring speed, titration speed, and equilibration time (Wang et al., 2008). The needle-like shape could induce abrasion of the mite, providing an effective physical control mechanism along with biocompatibility and antibacterial activity.

#### Other Inorganic Materials

Other mineral materials containing Ag, Ba, Cd, Co, Cu, F, I, Li, Mn, Mo, and Zn affect mite fecundity and egg viability (Boczek et al., 1984). However, the practical application of such materials as acaricides is unlikely because of economic or environmental concerns.

## CONCLUSIONS

Poultry red mite infestations are a serious problem in laying hens worldwide, and increasing infestations, along with acaricide resistance and pesticide toxicity to nontarget organisms and the environment, are problematic, even for other domestic fowl, pets, and other animals (including humans). Increasing interest in PRM control and eradication-related research has improved our understanding of its biology and ecology, prompting the proposal of various alternative control methods. Advances in microbiology have facilitated the development of effective vaccines, and the demand for natural products has led to the development of plant-derived compounds, natural enemies, growth regulators, and biopesticides as conventional pesticide alternatives. Although such strategies seek to reduce chemical-induced toxicity, many of these approaches still require testing and approval by governmental authorities before their registration for safe use and application, and may even require scaled-up preparation to reduce production costs.

The use of inorganic materials, including DE, silica-based products, kaolin clay, and talc, has received attention for several years as alternative strategies approved for the treatment of PRM. In several regions, only natural silica can be used on organic farms. The amorphous form of silica is considered harmful to the environment, animals, and human health. Silica-based inorganic materials are not poisonous and dehydrate the epicuticle of the PRM exoskeleton through the absorbent properties of silicon dioxide, causing desiccation of the mites. Many of these silica-based products are commercially available and widely used in Europe. The effectiveness against PRM varies between different silica-based products owing to differences in the absorption capacity of the particles, chemical composition, particle size, and specific surfaces. These physical properties have encouraged the development of new materials with similar or better properties to achieve mite control. In particular, increasing the water adsorption properties derived from porous structures in a material can lead to successful advances in inorganic materials for physically controlling red mites. Furthermore, these materials can have practical applications in many fields, including other pest insect management, without any approval required.
